# A Phylogeographic Description of *Histoplasma capsulatum* in the United States

**DOI:** 10.3390/jof9090884

**Published:** 2023-08-29

**Authors:** Ujwal R. Bagal, Lalitha Gade, Kaitlin Benedict, Victoria Howell, Natalie Christophe, Suzanne Gibbons-Burgener, Sara Hallyburton, Malia Ireland, Stephanie McCracken, Alison Keyser Metobo, Kimberly Signs, Kimberly A. Warren, Anastasia P. Litvintseva, Nancy A. Chow

**Affiliations:** 1 Mycotic Diseases Branch, Centers for Disease Control and Prevention, Atlanta, GA 30333, USA; 2 ASRT Inc., Atlanta, GA 30080, USA; 3 Kentucky Department for Public Health, Frankfort, KY 40601, USA; 4 Louisiana Department of Health, Baton Rouge, LA 70802, USA; 5 Wisconsin Department of Health Services, Madison, WI 53703, USA; 6 Indiana State Department of Health, Indianapolis, IN 46204, USA; 7 Minnesota Department of Health, St. Paul, MN 55101, USA; 8 Michigan Department of Health and Human Services, Lansing, MI 48933, USA; 9 Nebraska Department of Health and Human Services, Lincoln, NE 68508, USA; 10 Pennsylvania Department of Health, Wilkes-Barre, PA 18701, USA

**Keywords:** histoplasmosis, *Histoplasma capsulatum*, genome, clades, genomic, epidemiology

## Abstract

Histoplasmosis is one of the most under-diagnosed and under-reported endemic mycoses in the United States. *Histoplasma capsulatum* is the causative agent of this disease. To date, molecular epidemiologic studies detailing the phylogeographic structure of *H. capsulatum* in the United States have been limited. We conducted genomic sequencing using isolates from histoplasmosis cases reported in the United States. We identified North American Clade 2 (NAm2) as the most prevalent clade in the country. Despite high intra-clade diversity, isolates from Minnesota and Michigan cases were predominately clustered by state. Future work incorporating environmental sampling and veterinary surveillance may further elucidate the molecular epidemiology of *H. capsulatum* in the United States and how genomic sequencing can be applied to the surveillance and outbreak investigation of histoplasmosis.

## 1. Introduction

*Histoplasma capsulatum* is a thermally dimorphic fungus that can cause histoplasmosis when inhaled. It is non-contagious and affects humans and other mammals [1,2]. The fungus predominately lives in soil that is contaminated with bat droppings, thus suggesting bats as the potential natural reservoir of the fungus [3]. Numerous cases and outbreaks have been associated with exposure sites, such as caves and abandoned buildings where there is presence of bird or bat droppings. Additionally, infections are often linked to activities that disturb the soil such as mining and construction work [4,5,6]. Clinical presentation ranges from mild self-resolving to moderate pneumonia-like symptoms to a severe, life-threatening, disseminated disease. Histoplasmosis can affect healthy individuals or those with compromised immune systems. In the case of disseminated histoplasmosis, the infection can affect several organs including the lungs, bone marrow, skin, brain, and gastrointestinal tract [7,8].

In the United States, *H. capsulatum* is endemic to central and eastern states around the Ohio River Valley and the Mississippi River Valley [9]. It is estimated that 60–90% of the population living in this area has been exposed to the fungus [9]. However, disease surveillance is limited, with histoplasmosis being reportable to public health authorities in only 12 states [10]. Among the reported cases in 2019 (>1000), the high rate of hospitalization (54%) and death (5%) suggests that the actual number of cases is likely higher [11]. Furthermore, the systematic environmental surveillance of *H. capsulatum* is not conducted. Therefore, due to the under-detection of infections and limited surveillance, the true geographic distribution of *H. capsulatum* in the United States is poorly understood [12].

Based on morphology and pathogenic characteristics, the *Histoplasma* genus was thought to consist of three distinct varieties: *H. capsulatum*, *H. duboisii,* and *H. farciminosum* [13]. In 2003, Kasuga et al. utilized a genealogical concordance–phylogenetic species concept (GC–PSC) to classify *H. capsulatum* into eight clades: North American clades 1 and 2 (NAm 1 and NAm 2), Latin American clades A and B (LAm A and LAm B), Eurasian, Netherlands, Australian, and African, as well as a distinct lineage (H81) comprised of Panamanian isolates [14]. LAm A and LAm B clades, which comprised isolates from Mexico, Suriname, Guatemala, Brazil, and Argentina [14], exhibited the highest genetic diversity. Additionally, distinct genetic clusters of isolates reported from environmental, clinical, and naturally infected bat samples revealed a complex genetic structure within the Latin America population that included six subclades nested within the highly diverse LAm A and Lam B clades [15,16,17]. Furthermore, a few cases of LAm A, NAm 1, and NAm 2 clades co-occurred in the endemic areas of North America with different population dynamics [14,16]. More recently, Sepulveda et al. used genomic sequencing to classify *H. capsulatum* into five genetically distinct clades, of which four could be considered as species: NAm 1 (also referred to as the *H. mississippiense* species), NAm 2 (also referred to as the *H. ohiense* species), LAm A (also referred to as the *H. suramericanum* species), Panama lineage H81 (also referred to as the *H. capsulatum sensu stricto* species) and African [18]. In 2022, a new Indian lineage was reported [19]. However, it is important to note that these clades defined by genomic sequencing have not yet been accepted as valid species [20].

Whole-genome sequencing (WGS), compared with more traditional molecular typing methods, has proven to be a superior method for molecular surveillance and the epidemiology of infectious diseases [21]. Specifically, it allows for the detection of genome-wide polymorphisms that can be highly correlated with epidemiologic data and spatio-temporal spread [22]. WGS can also help trace transmission, identify the source of an outbreak, and elucidate the evolution of a pathogen. In the case of *H. capsulatum*, WGS helped reclassify the five distinct major clades that were previously phenotypically identified as three clades, demonstrating its high resolution and ability to refine our understanding of pathogen diversity.

Here, we utilize WGS to better describe *H. capsulatum* in the United States. We present the phylogeographic structure of *H. capsulatum* within the United States by utilizing clinical isolates obtained from a previous enhanced surveillance study of histoplasmosis patients from eight U.S. states [10].

## 2. Materials and Methods

### 2.1. Culture and DNA Extraction

*H. capsulatum* clinical isolates were received at the Mycotic Diseases Branch laboratory at the U.S. Centers for Disease Control and Prevention (CDC) for routine fungal identification as part of ongoing fungal disease surveillance. Upon arrival, species identification was conducted by sequencing the ITS2 region of the rDNA and then isolates were stored in 20% glycerol at −70 °C for further studies. Later, 48 isolates from this study were grown on brain–heart-infusion (BHI) agar at 25 °C for ≤10 days. Genomic DNA was extracted using the Qiagen DNeasy Blood and Tissue kit (Qiagen, Gaithersburg, MD, USA) according to the manufacturer’s instructions. All procedures were conducted in a Biosafety Level 3 laboratory.

### 2.2. Genomic Sequencing

Genomic libraries were constructed and barcoded using the NEBNext Ultra DNA Library Prep kit (New England Biolabs, Ipswich, MA, USA) for Illumina sequencing following the manufacturer’s instructions. Libraries were sequenced on the Illumina HiSeq 2500 platform (Illumina, San Diego, CA, USA) using the HiSeq Rapid SBS Kit v2 500 cycles. Raw sequence data were submitted to NCBI Sequence Read Archive (BioProject PRJNA868688).

### 2.3. Single-Nucleotide Polymorphism (SNP) and Phylogenetic Analysis

For the whole-genome SNP analysis, MycoSNP (v1.4) (https://github.com/CDCgov/mycosnp-nf), a reference-based SNP calling workflow was used [23]. The publicly available assembled genome of the *Histoplasma capsulatum* strain G217B (GenBank accession number: GCA_017607445.1) with 12 contigs and belonging to the NAm 2 clade was used as the reference. Additionally, sequences from SRR8347492(HC_7909), SRR6243656(CI_17), SRR6243645(3_11), SRR6243650(G186A), and SRR6243635(CI_19) were included in the analysis. With MycoSNP, the genome was masked for repeats using the nucmer command from mummer (v3.23) [24] and Bedtools (v2.30). The reference genome was indexed for downstream analysis [25,26]. Low-quality data trimming and filtering were performed using FaQCs (v 2.10) [27]. The trimmed reads were used for alignment using the BWA (0.7.17) MEM command [28]. Further, the aligned BAM files from each sample were pre-processed for variant calling using the genome analysis toolkit GATK (v 4.2.4.1) [29] with the haploid mode. GATK’s VariantFilteration tool was used to filter sites based on the filtering expression “QD < 2.0 || FS > 60.0 || MQ < 40.0”. Customized filtering criteria of minimum genotype quality <50, percentage alternate allele <0.80, and minimum depth of 10 were applied.

For the phylogenetic analysis, variant sites were concatenated, allowing for a maximum of 10% of samples with ambiguous nucleotides for selecting a site. A pairwise distance matrix and a neighbor joining tree (NJ) were created using MEGA 7 [30], and the maximum likelihood (ML) tree was constructed using FastTreeMP (v 2.1.11) [31] using the GTR nucleotide substitution model and a bootstrap analysis based on 100 replicates. A multi-dimensional scaling (MDS) technique was used (R function cmdscale) [32] to visualize the clustering pattern of these samples. A patristic distance matrix was used to construct an MDS plot of the complete tree as well as specific clades.

## 3. Results

Among the 48 total isolates, 39 (81%) case patients were male, 25 (52%) were aged 20–65 years, and 19 (40%) were immunosuppressed (Table 1). Most (*n* = 17, 34%) had a positive culture from bronchial specimens. Overall, the cases resided in eight U.S. states (Indiana [*n* = 2], Kentucky [*n* = 4], Louisiana [*n* = 1], Michigan [*n* = 21], Minnesota [*n* = 13], Nebraska [*n* = 1], Pennsylvania [*n* = 1], and Wisconsin [*n* = 5]), and most were from Michigan (44%), Minnesota (27%), and Wisconsin (10%)).

**Figure 1:**
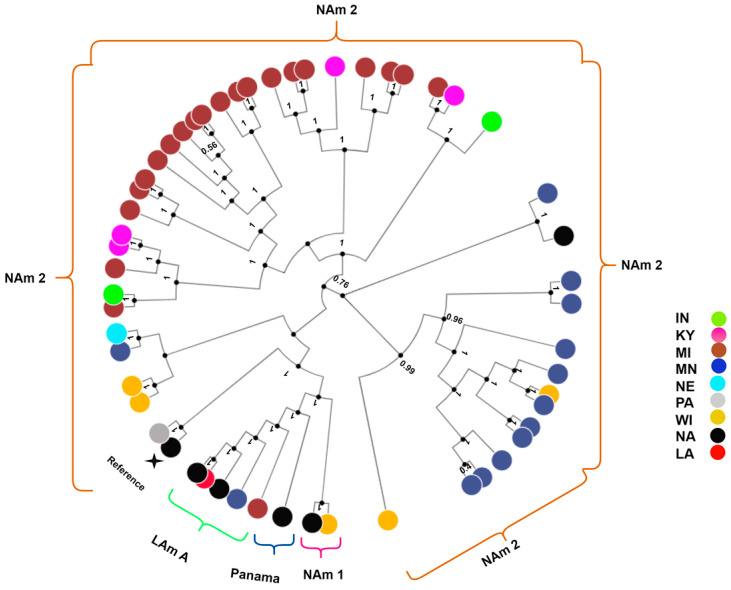
Phylogenetic analysis of *H. capsulatum* isolates from eight U.S. states. The ML tree includes 54 isolates. Node color is based on the associated U.S. states where the patient resided. The following are the eight states: Indiana (IN), Kentucky (KY), Michigan (MI), Minnesota (MN), Nebraska (NE), Pennsylvania (PA), Wisconsin (WI), Louisiana (LA). Not available (NA).

Genomic sequencing and SNP analysis identified 1,969,979 variant sites, which were used for constructing an NJ and ML phylogenetic tree. The phylogenetic analysis revealed that the isolates formed clades as previously described for *H. capsulatum* (Figure 1). Specifically, 44 (92%) samples clustered with the NAm 2 clade samples SRR6243656 and the reference genome (Figure 1). Three (6%) isolates clustered with the LAm A clade. One (2%) isolate clustered with the NAm 1 clade. Isolates from the LAm A and NAm 2 clades were separated by ≤346,613 SNPs and isolates from the NAm1 and NAm 2 clades were separated by ≤600,854 SNPs.

**Figure 2:**
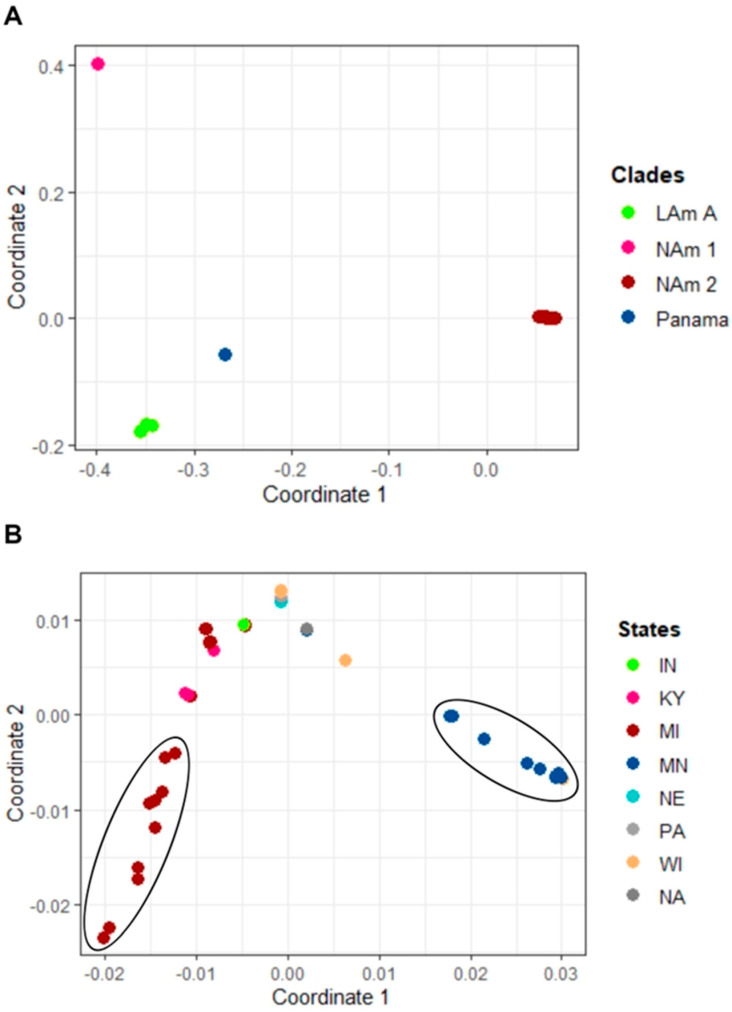
Multi−dimensional (MDS) plot of *H. capsulatum* isolates. (**A**) MDS plot of the patristic distance with x axis being dimension 1 and y axis dimension 2. The plot which includes all isolates revealed four distinct clades. Most (92%) of the samples clustered with the NAm 2 clade, three with the LAm A clade, one with the NAm 1 clade, and none clustered with the Panama control sample. (**B**) MDS plot of isolates from the NAm 2 clade. Two separate clusters of isolates belonging to MI and MN were observed. The following are the eight states: Indiana (IN), Kentucky (KY), Michigan (MI), Minnesota (MN), Nebraska (NE), Pennsylvania (PA), Wisconsin (WI), Louisiana (LA). Not available (NA).

Additionally, MDS analysis showed similar findings to the phylogenetic tree whereby distinct clusters of NAm1, NAm2, and LAm A clades were observed (Figure 2A). The NAm 2 clade comprised two clusters whereby isolates were primarily grouped by state. One cluster contained 11 isolates; 10 were from cases from Minnesota and 1 from a case from Wisconsin (Figure 2B). A second cluster contained 10 isolates from cases from Michigan. The remaining eleven cases from Michigan, four from Kentucky, two from Indiana, four from Wisconsin, two from Minnesota, one from Nebraska, and one from Pennsylvania clustered together in the third cluster. The reference sample was the most distant isolate within the NAm2 clade (not included in the MDS plot).

## 4. Discussion

The phylogeographic structure of *H. capsulatum* in the United States is poorly understood. Modeling studies have predicted potential shifts in the geographic distribution of *H. capsulatum* and other environmental fungal pathogens within the United States [33]. As previously observed, the expanding presence of fungal pathogens such as *H. capsulatum* in newer geographic areas could be attributed to changes in their ecological niche, alterations in the behavior of their natural reservoirs, and dispersers [34]. Additionally, soil contamination via the guano of birds and bats is believed to play a crucial role in the dispersal of *H. capsulatum* [16]. It has been proposed that bats, which are susceptible to *Histoplasma* infection and can cover extensive distances, serve as both a natural reservoir and carrier, facilitating the dispersal of *Histoplasma* in suitable environments and introducing the fungus to previously unaffected areas [2,15]. Notably, as human social behavior evolves and climate shifts occur, conditions are becoming favorable for the fungus and bats, thereby contributing to the expansion of the habitat [17,34]. To improve our understanding of the lineages and clade-specific genetic variations of *H. capsulatum* in the United States, we evaluated 48 histoplasmosis cases from eight U.S. states. By performing a whole-genome analysis on the associated isolates, we leveraged the power of WGS, which is recognized as a highly effective molecular epidemiologic tool that provides much greater epidemiologically relevant resolution than classical genotyping methods like MLST.

Our analysis revealed a single NAm 1 clade isolate from Wisconsin, which is not unexpected given that the NAm 1 clade has been previously reported in North America, both in the United States and Canada [14,35]. Likewise, we also found three isolates from Minnesota, Michigan, and Louisiana that grouped with the LAm A clade, which has also been previously reported in the United States and Canada [16,35]. The identification of isolates from the LAm A clade underscores the necessity for the additional sampling of environmental isolates to understand the true geographical range of *H. capsulatum*. Since environmental isolates were not available, our study was limited to the analysis of clinical isolates only.

Most isolates in this study belonged to the NAm 2 clade, a finding that is consistent with previous work [14]. Despite belonging to the same clade, isolates showed a high degree of SNP differences (maximum of 60,000 SNPs), highlighting high within-clade genetic diversity (Supplementary Figure S1). NAm 2 is amongst the oldest *H. capsulatum* clades, which is distant from other clades, and is hypothesized to have emerged between 3.2–13 million years ago [14]. *H. capsulatum* is well known for its genetic complexity and the role played by geographical expansion in the creation of new lineages with notable phenotypic and virulence differences [14]; for example, the prevalence of the extensive genetic variation of *H. capsulatum* in Latin America has been documented previously [36]. A possible reason for large intra-clade diversity within the NAm 2 clade could be due to recombination and changes in selection pressures because of the expanding geographic boundaries, forcing the fungi to rapidly adapt to varying environmental changes [34,37].

Within NAm2, we identified two genetically distinct clusters, with isolates primarily grouping by state. Most cases from Minnesota were found in one cluster, while those from Michigan were in another cluster, with a few exceptions where isolates from Michigan clustered with those from other states. Moreover, one isolate from Wisconsin was found in the Minnesota cluster. This could be due to an exposure occurring in Minnesota when the patient resided in Wisconsin. It was not possible to determine whether cases were locally acquired or travel-related such as from neighboring or visiting states. For future studies, interdisciplinary approaches that tap into environmental samples or samples from veterinary surveillance that have robust epidemiologic data may prove useful.

Regarding the future applications of WGS for the genomic surveillance and epidemiology of histoplasmosis, there may be a potential role for this technology as performed for *Coccidoides immitis.* WGS has proven to be an effective method to identify locally acquired Valley fever due to the well-defined phylogeographic structure of *C. immitis*. Specifically, it is possible to identify cases of locally acquired Valley fever in Washington and delineate between exposures in Washington and California [38]. Histoplasmosis, like Valley fever, also has endemic and non-endemic areas. However, it is unknown whether *H. capsulatum* has a strong phylogeographic structure as described for *C. immitis*. Therefore, to understand whether WGS can be used to help determine locally acquired cases of histoplasmosis, a more robust characterization of the phylogeographic population structure is needed. Here, we show evidence that this may be possible for some endemic states such as Minnesota and Michigan, but further studies are needed that incorporate environmental sampling and comprehensive travel history to confirm this.

Overall, we employed WGS to investigate the prevalence of *Histoplasma* lineages in the United States. Our findings shed light on the phylogeographic structure of this significant pathogen and raise questions regarding the potential utility of WGS for the genomic epidemiology of histoplasmosis.

## Figures and Tables

**Table 1:** Associated case characteristics for *Histoplasma capsulatum* isolates collected from eight U.S. states.

	Count	Percentage (%)
**Sex**		
Male	39	81
Female	9	19
Total	48	100
**Age Group**		
<21	6	13
≥21 & <65	25	52
≥65	17	35
Total	48	100
**Immunosuppressed cases**		
Indiana	1	5
Kentucky	3	16
Michigan	6	32
Minnesota	8	42
Wisconsin	1	5
Total	19	100
**Specimen Source**		
Sputum	4	8
Lymph node	4	8
Bronchial specimen	17	35
Lung tissue	3	6
Blood	12	25
Bone marrow	3	6
Others *	5	10
Total	48	100
**States**		
Indiana	2	4
Kentucky	4	8
Louisiana	1	2
Michigan	21	44
Minnesota	13	27
Nebraska	1	2
Pennsylvania	1	2
Wisconsin	5	10
Total	48	100

* Others included fluid synovial, wound, tissues (arm and lungs), and unknown sample source.

## Data Availability

Not applicable.

## Supplementary Materials

The following supporting information can be downloaded at: https://www.mdpi.com/article/10.3390/jof9090884/s1, Figure S1: Neighbor-joining tree of *H. capsulatum* isolates showing SNP differences. File S1: S1_Hcapsulatum_isolates_metadata.xlxs.
