# Supplementary material for: A Phylogeographic Description of Histoplasma capsulatum in the United States
Source: J Fungi (Basel). 2023 Aug 29;9(9):884. doi: 10.3390/jof9090884 (PMC10532573; doi:10.3390/jof9090884)
Supplement: Supplementary file 1 [file Supplementary_Figure_S1legend.pdf › Supplementary_Figure_S1legend.pdf]

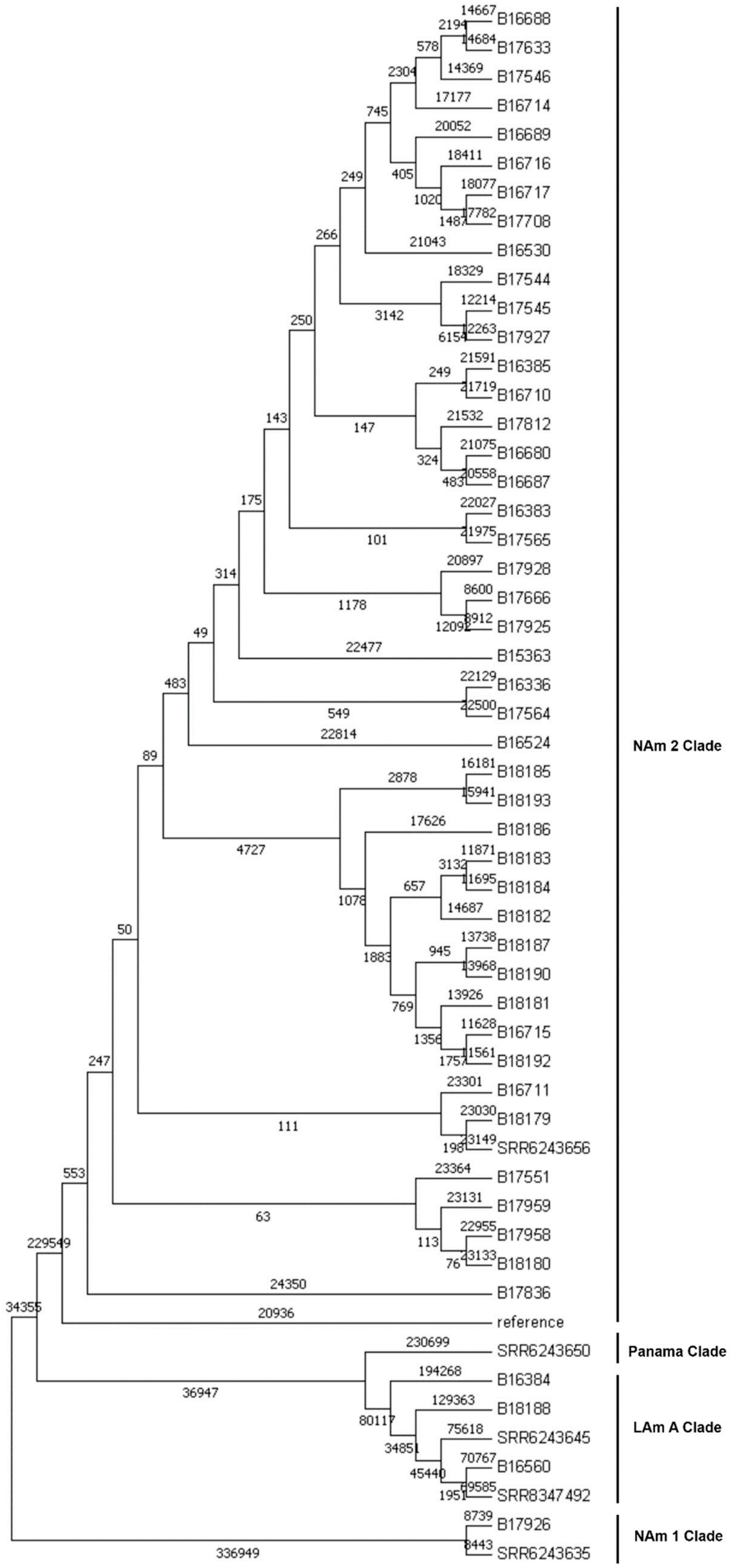

Supplementary Figure S1: Neighbor-joining tree of *H. capsulatum* isolates showing SNP differences.

The neighbor-joining tree includes 48 isolates and 5 control samples along with a reference belonging to the NAm 2 clade. Most of the isolates clustered with the NAm 2 clade control sample, while three isolates clustered with the LAm A clade, and one with the NAm 1 clade. The values present on the tree branches are SNP values.
